# Hydrophilic silver nanoparticles with tunable optical properties: application for the detection of heavy metals in water

**DOI:** 10.3762/bjnano.7.157

**Published:** 2016-11-09

**Authors:** Paolo Prosposito, Federico Mochi, Erica Ciotta, Mauro Casalboni, Fabio De Matteis, Iole Venditti, Laura Fontana, Giovanna Testa, Ilaria Fratoddi

**Affiliations:** 1 Department of Industrial Engineering, INSTM and CIMeR, University of Rome Tor Vergata, Rome, V. del Politecnico 1, 00133, Italy; 2 Department of Chemistry, University of Rome Sapienza, Rome, P.le A. Moro 5, 00187, Italy

**Keywords:** heavy metal sensor, nickel (II), optical materials, optical sensors, silver nanoparticles

## Abstract

Due their excellent chemo-physical properties and ability to exhibit surface plasmon resonance, silver nanoparticles (AgNPs) have become a material of choice in various applications, such as nanosensors, electronic devices, nanobiotechnology and nanomedicine. In particular, from the environmental monitoring perspective, sensors based on silver nanoparticles are in great demand because of their antibacterial and inexpensive synthetic method. In the present study, we synthesized AgNPs in water phase using silver nitrate as precursor molecules, hydrophilic thiol (3-mercapto-1-propanesulfonic acid sodium salt, 3MPS) and sodium borohydride as capping and reducing agents, respectively. The AgNPs were characterized using techniques such as surface plasmon resonance (SPR) spectroscopy, dynamic light scattering (DLS), zeta potential (ζ-potential) measurements and scanning tunneling microscopy (STM). Further, to demonstrate the environmental application of our AgNPs, we also applied them for heavy metal sensing by detecting visible color modification due to SPR spectral changes. We found that these negatively charged AgNPs show good response to nickel (II) and presented good sensibility properties for the detection of low amount of ions in water in the working range of 1.0–0.1 ppm.

## Introduction

Nanomaterials have become very popular in the last years in many fields because of their unique electronic, optical, magnetic and photocatalytic properties and for their large surface-to-volume ratio, which allows very good interaction with the external environment [1–6]. These properties can be exploited in the field of sensors for specific analytes [7–10]. In particular, given their flexible and easy preparation, large specific surface area, and surface plasmon resonance (SPR) properties, metal nanoparticles are excellent candidates for a wide variety of applications ranging from catalysis [11–12], energy [13–15], optoelectronics [16–17] and biomedicine [18] to sensors [19–20]. A very promising field of application is chemical sensing, which includes gas detection, safety and environmental monitoring [21–25].

Moreover, the need for developing highly sensitive and selective sensors for the detection of very low amounts of heavy metal ions from biological and environmental samples has greatly increased [26]. The metal-ion toxicity depends on their physical state, chemical form as well as the oxidation state. Many groups have studied ﬂuorescent chemosensors with ﬂuorescence enhancement and quenching for the detection of metal ions [27–32].

The most studied nanoparticles are those based on gold, however many studies have revealed that a suitable functionalization of silver nanoparticles can significantly improve their sensing performance [17–22]. The presence of specific functionality on the particle surface can increase the adsorption of the analytes, resulting in an enhancement of sensitivity or selectivity. An interesting application of functionalized AgNPs is reported by Contino et al. [33] where synthesized AgNPs were capped by tyrosine and the heavy metal sensing performance was measured by observing the shift of SPR band. In this case the low detection limit for Cu(II) is attributed to the high quality of the AgNPs.

From the perspective of optimizing the properties of metal nanoparticles, research groups have focused on the control of the size and shape of nanoparticles [34–35], which is crucial in tuning their physical, chemical and optical properties [36–38].

Electrochemical, photochemical, sonochemical and chemical reduction methods can be used for the synthesis of metal nanoparticles [39–43]. On the other hand, the wet reduction is the most common synthetic technique for the fabrication of metal nanoparticles, using sodium borohydride as a reducing agent and thiols as a capping agent to prevent nanoparticles aggregation [44–46].

In the present article, we report the fabrication and characterization of AgNPs stabilized by sodium 3-mercaptopropane sulfonate (AgNP-3MPS). A new preparation was optimized to improve the optical sensing performances based on SPR spectral changes. The AgNP-3MPS nanoparticles have been tested as optical sensors in water solution towards different metal ions at concentration of 1 ppm at room temperature. Our system showed sensibility mainly to nickel (II) ions. For this type of ion, we tested the sensitivity as a function of the ion concentration in the range 1.0–0.1 ppm and we estimated a limit of detection (LOD) of 0.3 ppm.

## Results and Discussion

### Silver nanoparticle synthesis

**Scheme 1:**
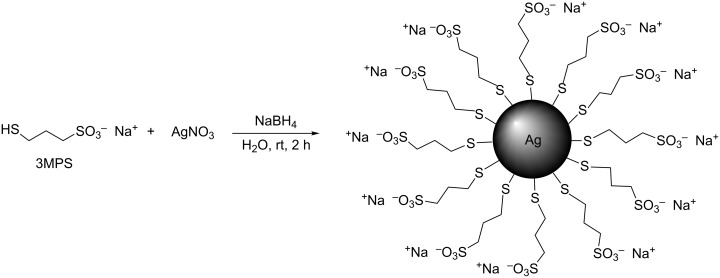
Synthetic scheme of AgNP-3MPS nanoparticles (synthesis a).

AgNPs were synthesized by wet reduction, using sodium borohydride as a reducing agent and 3-mercapto-1-propanesulfonic acid sodium salt as a capping agent. The water solubility of the thiol induces a strongly hydrophilic character with respect to the nanoparticles, which present the sulfonate groups on the surface [47]. The presence of these negatively charged groups also guarantees the stability of the colloids over time, as was confirmed by the ζ-potential measurements [48–49]. Moreover, to improve the monodispersity of the AgNPs two different synthetic approaches were used. In the first one (synthesis a) the procedure was developed in analogy with previous work [22] and is reported in Scheme 1. This step leads to the hydrophilic character of silver nanoparticles.

**Figure 1:**
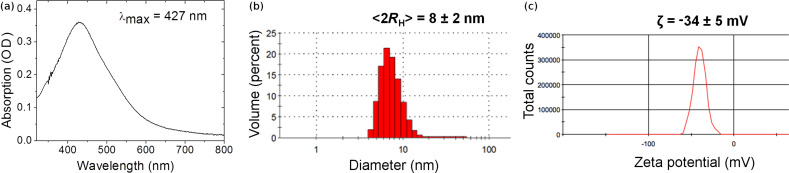
Characterization of AgNP-3MPS nanoparticles in H_2_O (synthesis a): (a) UV–vis spectrum; (b) DLS measurements: <2*R*_H_> = 8 ± 2 nm; (c) ζ-potential measurements: ζ = −34 ± 5 mV.

At the end of the synthesis and purification, the reaction product was analyzed by UV–vis spectroscopy to confirm the presence of AgNPs by means of the measurement of the SPR spectra with a peak at 425 nm in the absorption spectrum, as shown in Figure 1a. The full with at half maximum (FWHM) was found to be about 170 nm. The DLS data are reported in Figure 1b,c. They show the diameter and ζ-potential of Ag-3MPS NPs obtained after the purification of the crude reaction by centrifugation in H_2_O. Here we obtained well-dispersed AgNPs with an average diameter (<2*R*_H_>) of 8 ± 2 nm and a ζ-potential of −34 ± 5 mV.

The nanoparticle diameter measured in water solution and in dry conditions are statistically different because in the water dispersion the hydrodynamic diameter is is measured and includes solvent layers. This therefore creates a potential on the surface of the particles (the ζ-potential) that induces negative charges on AgNPs surface due to sulfonate groups.

The sensitivity of the AgNPs to heavy metal ions was tested in water solution by monitoring the optical absorption changes in the presence of metal ions. A weak dependence of the peak wavelength as a function of the ion concentration was found. However, the large broadening of the SPR band and the high background of the absorption hindered the observation of a net effect of the presence of the ion. In this regard, since our main goal was the exploitation of the possible changes of the SPR band as a function of the external environment, a sharp and narrow absorption band was mandatory. For these reasons, we tuned and optimized a second, modified procedure (synthesis b) for the synthesis of the AgNPs. In this case, the molar ratio between silver nitrate, sodium borohydride and 3-MPS was varied. The reaction was conducted at 3 °C and the final purification was avoided in order to prevent possible aggregation with a consequent broadening of the SPR band. For these reasons, we worked with very dilute AgNP solutions to minimize the residual unreacted chemical products. In particular, the concentration of the 3MPS was optimized to the ratio AgNO_3_/3MPS 1:0.1 to ensure good coverage of the particles with 3MPS molecules, and thus increase the sensitivity of the sensor as much as possible.

**Figure 2:**
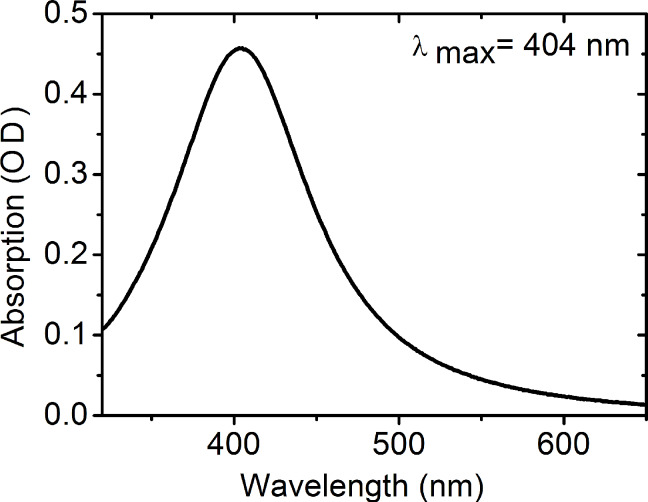
Absorption spectrum of AgNP-3MPS nanoparticles (synthesis b). The SPR exhibits a sharp peak at 404 nm with a FWHM of 92 nm.

Figure 2 shows the absorption spectra of the AgNP-3MPS solution. It shows a sharp peak at 404 nm. Its FWHM is 92 nm instead of 170 nm as obtained by the first synthetic method (synthesis a). It should be noted that the FWHM of the AgNP solution without capping agent (not reported) was about 60 nm. The broadening of about 30 nm can be ascribed to the surface functionalization of the AgNPs with 3MPS.

**Figure 3:**
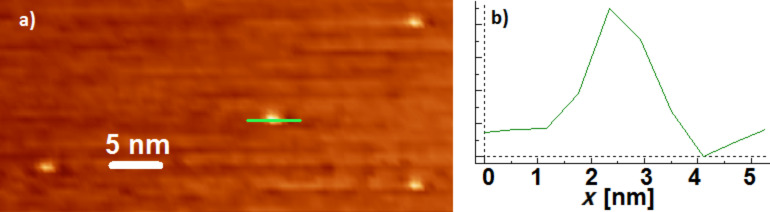
a) STM morphology measurements of AgNP-3MPS (synthesis b) and b) the height profile of one nanoparticle.

We characterized the AgNP-3MPS nanoparticles via STM spectroscopy. Figure 3a,b shows the morphology and the profile of the nanoparticles, respectively. We estimated the average diameter using about thirty nanoparticles with a resulting value of 2.5 ± 0.3 nm.

**Figure 4:**
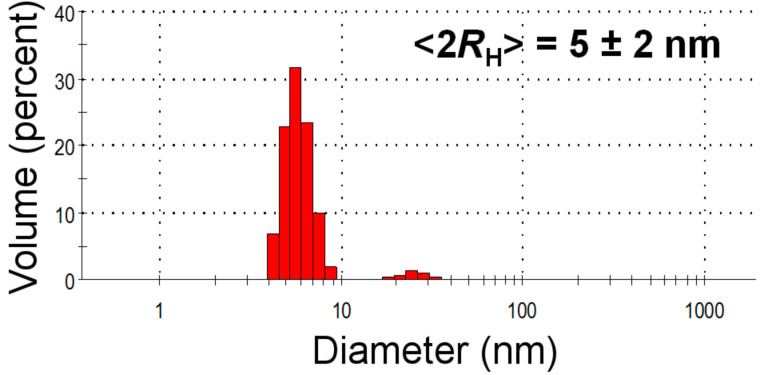
DLS of the AgNP-3MPS solution (synthesis b). The hydrodynamic diameter was <2*R*_H_> = 5 ± 2 nm.

Figure 4 shows the results from the dynamic light scattering (DLS) measurement on the AgNP-3MPS material (synthesis b). The distribution indicates good monodispersity of the NPs and an average diameter of <2*R*_H_> = 5 ± 2 nm, in good agreement with the STM analysis.

**Figure 5:**
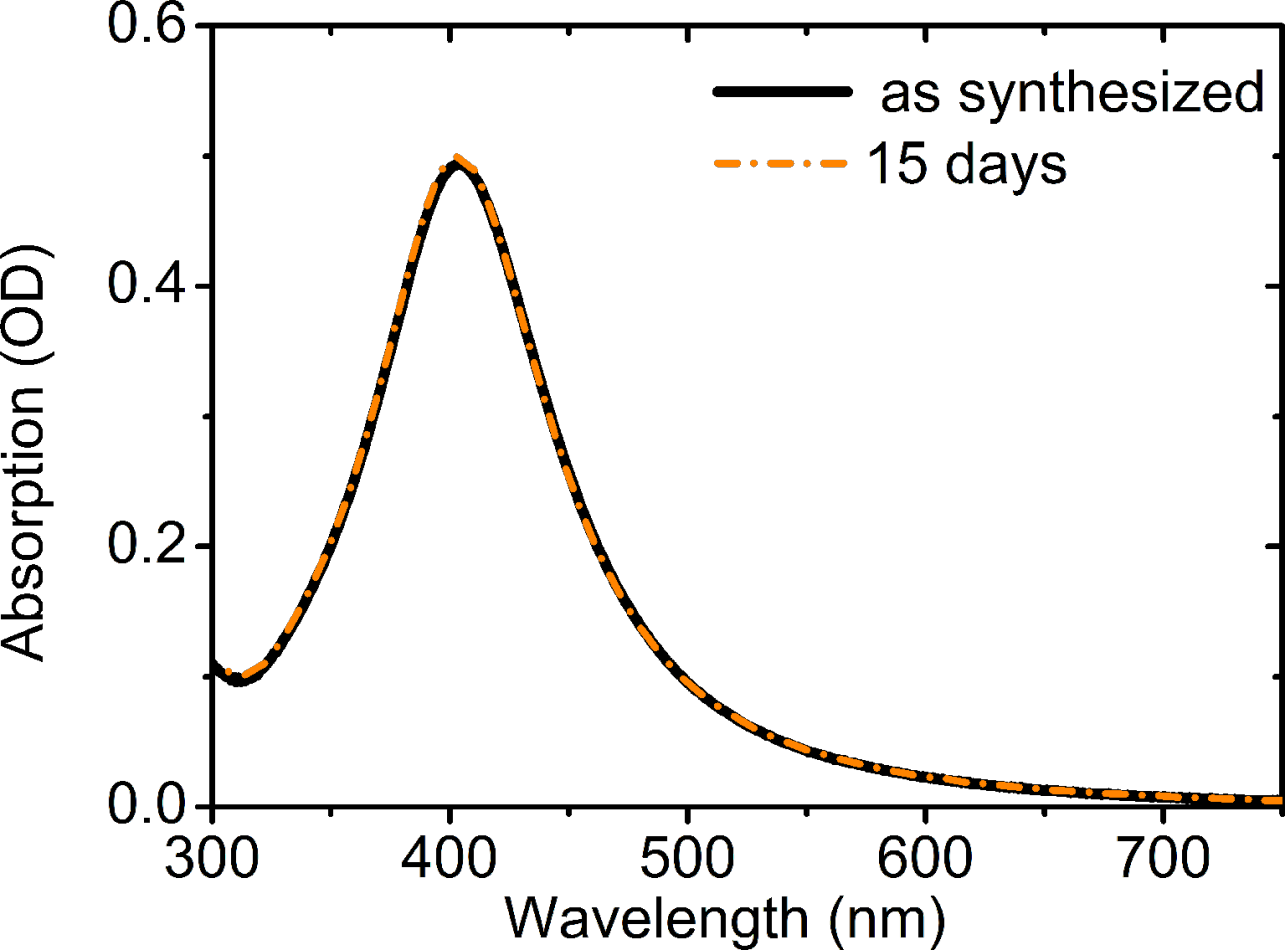
SPR absorption spectroscopy of the AgNP-3MPS solution (synthesis b) taken immediately after the synthesis and after 15 days, indicating good long term stability.

The SPR absorption was monitored as a function of the time in order to check the long term stability of the NPs. As shown in Figure 5, the solution presents the same optical spectra 15 days after the preparation, indicating that no aggregation of the nanoparticles occurred.

### Metal ion detection

We investigated the optical sensing properties of the AgNP-3MPS material via absorption spectroscopy. Each solution of AgNP-3MPS/ion was prepared in spectroscopic cuvettes that were carefully cleaned to avoid contamination. To a specific amount of AgNPs contained in a fixed volume of water, a fixed volume of water was added containing the ions with the specific final concentration. After the mixing, we waited for five minutes before measuring the absorption spectra in order to allow for the complete interaction with the NPs.

**Figure 6:**
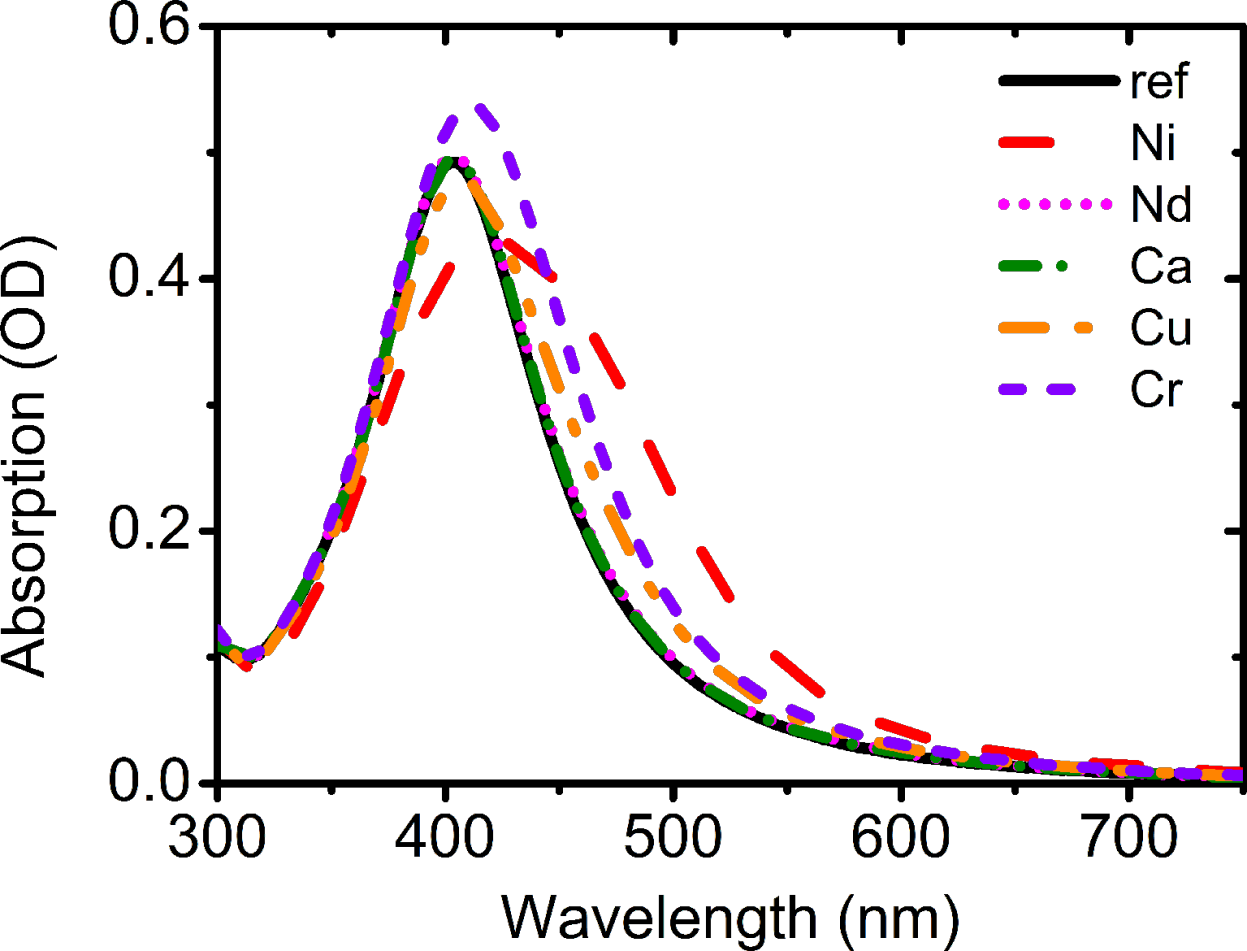
Absorption spectra of the AgNP-3MPS (synthesis b) solution with 1 ppm ion concentration.

The sensitivity of the AgNPs-3MPS system was tested with different metal ions: Ni^2+^, Cr^3+^, Nd^3+^, Cu^2+^ and Ca^2+^. We found different behavior for the various ions. In Figure 6 the SPR bands are reported relative to all the tested ions for a fixed value of the concentration (1 ppm).

The system shows a good response to Ni^2+^ since the absorption spectrum is strongly different with respect to that obtained from the solution without ions (reference). With the Cr^3+^ ions, only a small shift in the peak wavelength and an increase in the intensity is observed, while for the Ni^2+^ ions, a larger shift, a broadening and a reduction of the maximal value were detected in the plasmonic feature. The presence of Cu^2+^, Nd^3+^ and Ca^2+^ on the contrary does not produce any substantial modifications in the plasmonic absorption neither in the intensity nor in the position and shape.

**Figure 7:**
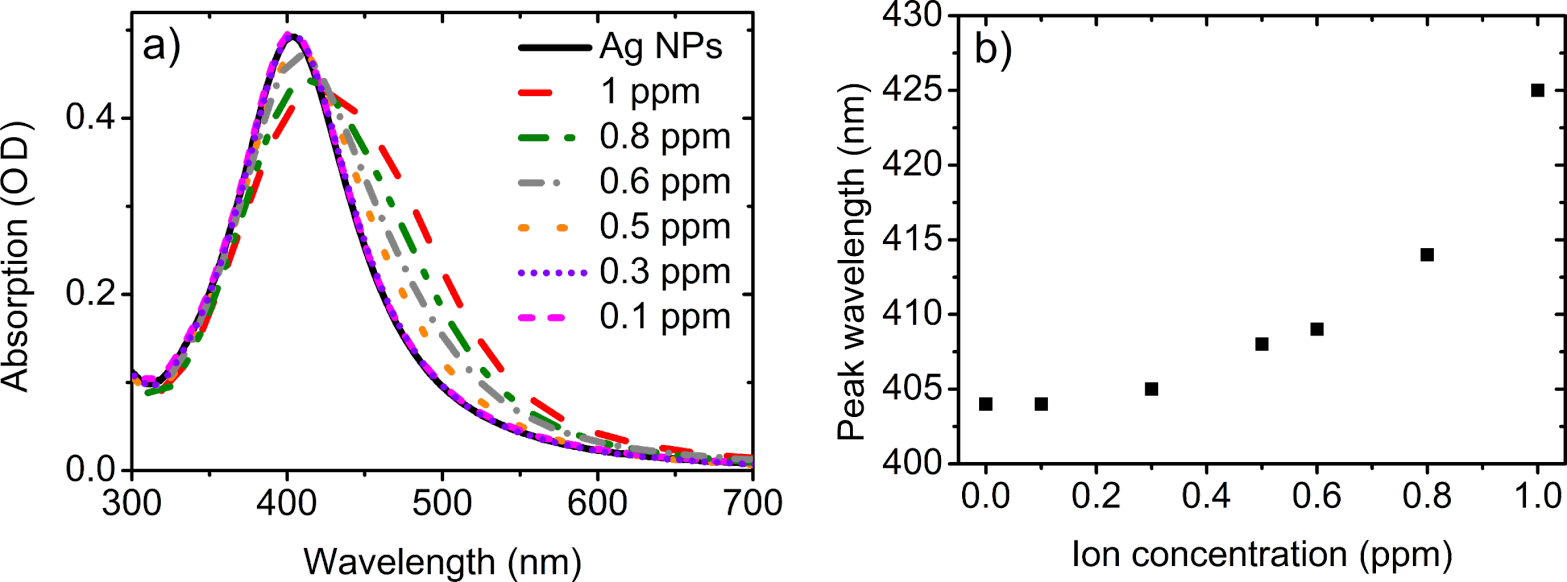
a) Absorption spectra of the AgNP-3MPS system (synthesis b) as a function of the Ni^2+^ concentration. b) Peak absorption wavelength as a function of the ion concentration.

To quantitatively test the properties of the NPs we performed absorption measurements as a function of concentration of nickel ions. Figure 7a shows the optical absorption spectrum of the AgNP-3MPS solution with nickel for different ion concentrations in the range 1.0–0.1 ppm. Figure 7b presents the maximum wavelength of the absorption peak as a function of the concentration of metal ions.

**Figure 8:**
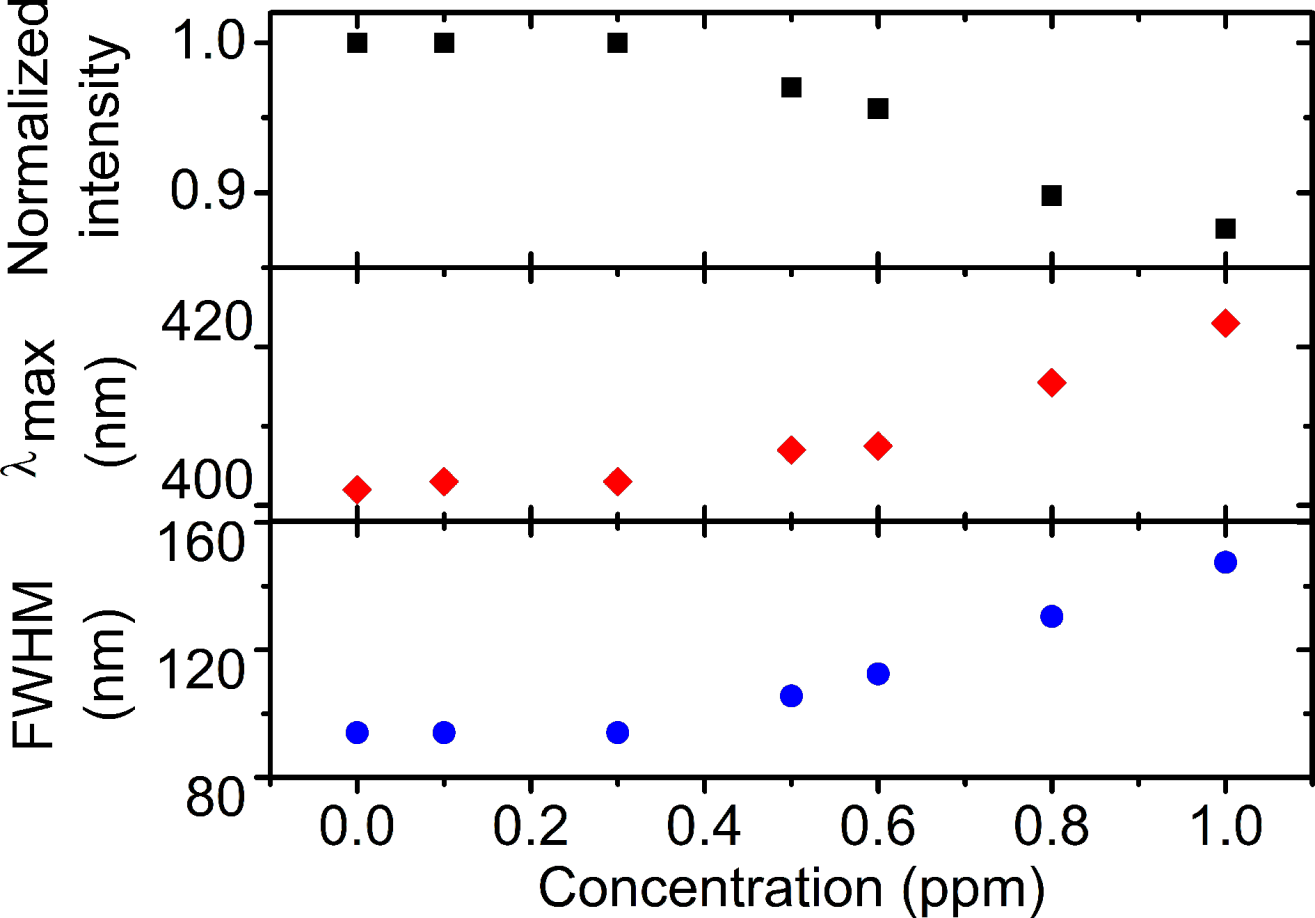
Normalized intensity, absorption maximum (λ_max_) and FWHM as a function of the nickel ion concentration.

In Figure 8 we report the normalized intensity, the peak wavelength and the FWHM of the absorption spectra obtained with different nickel ion concentrations (from 0–1 ppm). The reduction of intensity with the increase of the ion concentration can be ascribed to the reduction of the apparent number of NPs due to aggregation induced by the Ni^2+^ ions. The red shift and broadening of the SPR peak is a clear indication of the increase of the average size of the NPs and their size distribution and is fully compatible with the aggregation hypothesis.

At high ion concentrations (higher than 1 ppm), the shape of the SPR is sharper than that of the 1 ppm concentration and does not present sensitivity to different ion concentrations. This is probably due to the ratio between the number of ions and the number of silver nanoparticles. In the ion concentration range from 1 to 25 ppm, the number of ions exceeds the number of nanoparticles at least by a factor of 1000. Under these conditions, small changes of the number of ions do not affect the SPR band since all the external thiols present on the NP surface are saturated. On the other hand, for lower ion concentrations in the range 0.1–1 ppm the number of ions for each NP is on the order of a few tens and therefore a decrease in their number can strongly affect the optical properties of the solution. We can thus conclude that for high ion concentrations, the NPs are completely screened and a positive stabilization of the colloidal suspension occurs. In the intermediate region (0.3 to 1 ppm) the number of ions is comparable with the number of thiols surrounding the metal NPs and aggregation between NPs can easily occur. Finally, in the region below 0.3 ppm, the ions do not cause aggregation, but only a slight shift of the position of the surface plasmon.

**Figure 9:**
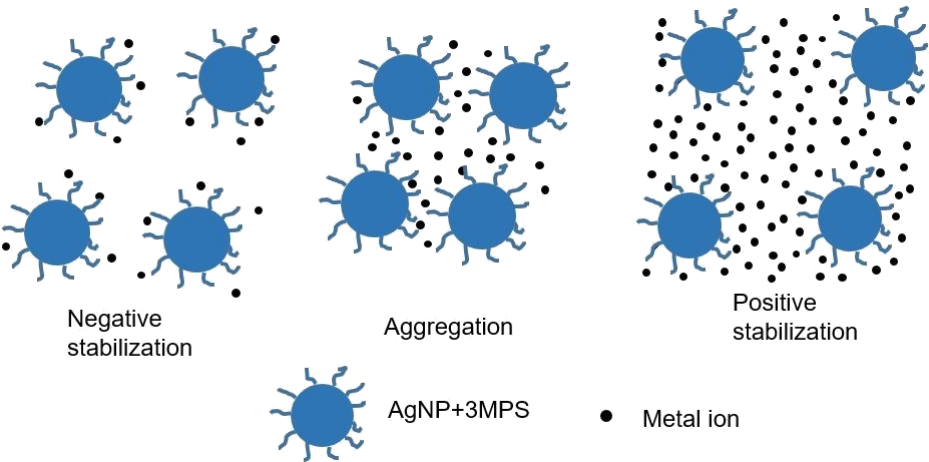
Model of the interaction of AgNP-3MPS and metal ions*.*

On the basis of these considerations, we propose a simple model to explain the observed behavior reported in Figure 9. We estimated the concentration of the AgNP-3MPS solution, the average number of surface thiols present for each NP, and the number of nickel ions for each concentration. For a concentration greater than 2 ppm, the value of the ratio (number of ions/number of NPs) is in the range 100–200. In this case, the metal ions are able to totally screen the NP signal, since the sulfonate groups of the 3MPS on a single nanoparticle are on the order of a few tens. Under this condition, a positive stabilization occurs. When the number of metal ions decreases to a few tens (and is therefore is comparable with the number of available surface thiols) the positive shell is not dense enough to totally screen the nanoparticle and aggregation occurs since the ions themselves can act as bridges between adjacent AgNPs. Finally, when the number of metal ions is lower than the number of surface thiols, the metal ions do not cause aggregation, but the only effect is a slight shift of the SPR peak wavelength position.

## Conclusion

In this work AgNP-3MPS nanoparticles with an average diameter of 2.5 ± 0.3 nm were synthetized with a narrow distribution range and negative charge on the surface. These particles were fully characterized by means of different techniques, such as STM, DLS and UV–vis spectroscopy. The SPR signal of the AgNP-3MPS sample in water solution demonstrated a spectral response to the presence of metal ions. This showed a sensitivity and selectivity to Ni^2+^ ions with a sensitivity as low as 0.3 ppm. A quantitative determination of these species in the concentration range of 0.3–1.0 ppm was provided. This approach seems very promising for the development of an analytical sensor system based on optical methods to detect metal ions in actual water samples.

## Experimental

### Materials and sample preparation

Silver nitrate (AgNO_3_, Sigma-Aldrich, 99.5%), sodium borohydride (NaBH_4_, Sigma-Aldrich, 98%) and 3-mercapto-1-propanesulfonic acid sodium salt (C_3_H_7_S_2_O_3_Na, 3MPS, Sigma-Aldrich, 98%) were used as received. Deionized water (electrical conductivity less than 1 μΩ/cm at room temperature) was obtained from a Millipore Milli-Q water purification system.

Synthesis a: silver NPs (AgNPs) were prepared at room temperature in a single phase system, following a procedure reported elsewhere [22,47,50]. AgNP-3MPS nanoparticles were prepared using a Ag/3MPS molar ratio of 0.25:1. In a typical synthesis, 0.100 g of AgNO_3_ were dissolved in 10 mL of deionized water and a solution of 3MPS in deionized water (0.840 g in 10 mL) was added; the mixture was maintained under argon flux for deoxygenating the reactions and vigorous stirring for 10 min at room temperature. Finally, NaBH_4_ solution in deionized water (0.223 g in 10 mL) was added dropwise. The reaction mixture was allowed to react for 2 h and the obtained suspension was centrifuged with deionized water several times (10 min, 15 000 rpm).

Synthesis b: sodium borohydride and silver nitrate solution (in deionized water) was mixed with concentration ratio NaBH_4_/AgNO_3_ 2:1. The NaBH_4_ solution was cooled to 3 °C under vigorous stirring, then the AgNO_3_ solution was added dropwise at approximately one drop per second. The volume ratio of the two solutions was NaBH_4_/AgNO_3_ 15:1. When all of the AgNO_3_ solution was added, the stirring was stopped. Then the 3MPS was added to reach the final ratio of AgNO_3_/3MPS 1:0.1. While adding the 3MPS, the solution was stirred for few seconds, then the AgNP-3MPS solution was stored at 4 °C.

For samples obtained with both synthesis methods, UV–vis spectra were run with a Varian Cary 100 Scan UV–visible spectrophotometer and Perkin-Elmer Lambda 19 from water suspensions. A Malvern Zetasizer Nanoseries instrument (Malvern, UK) equipped with a 10 mW HeNe laser at a 632.8 nm wavelength was used to obtain the DLS measurements on the AgNP aqueous suspensions (0.2 mg/mL) at *T* = 25.0 ± 0.2 °C. The correlation functions were collected at 90° relative to the incident beam, and delay times from 0.8 µs to 10 s were explored. Non-negative least-squares (NNLSs) [51] or CONTIN [52] algorithms, supplied with the instrument software, were used to fit the correlation data. The average hydrodynamic radius of the diffusing objects was calculated, as reported in previous studies [53–54]. The ζ-potential was calculated from the measured electrophoretic mobility by means of the Smolukovsky equation [55]. The scanning tunneling microscope (Tops System, WA Technology) consists of a UHV attachment with an antivibration stacking and a piezoelectric tube with 2 mm maximum scanning area for the tip movement. The lateral resolution of the microscope is ±1 Å and the accuracy in the lateral displacement is ±0.05 Å. Tungsten tips were chemically etched in a solution of NaOH and glycerol.
